# Potential premalignant status of gastric portion excluded after Roux en-Y gastric bypass in obese women: A pilot study

**DOI:** 10.1038/s41598-019-42082-4

**Published:** 2019-04-03

**Authors:** Graziela Rosa Ravacci, Robson Ishida, Raquel Suzana Torrinhas, Priscila Sala, Natasha Mendonça Machado, Danielle Cristina Fonseca, Gisele André Baptista Canuto, Ernani Pinto, Viviane Nascimento, Marina Franco Maggi Tavares, Paulo Sakai, Joel Faintuch, Marco Aurelio Santo, Eduardo Guimarães Hourneaux Moura, Ricardo Artigiani Neto, Angela Flávia Logullo, Dan Linetzky Waitzberg

**Affiliations:** 1 Departamento de Gastroenterologia, Laboratorio Metanutri (LIM35), Faculdade de Medicina FMUSP, Universidade de Sao Paulo, Sao Paulo, SP Brazil; 2 Hospital das Clinicas HCFMUSP, Faculdade de Medicina, Universidade de Sao Paulo, Sao Paulo, SP Brazil; 3 Departamento de Quimica Analitica, Instituto de Quimica, Universidade Federal da Bahia, Salvador, BA Brazil; 4 Faculdade de Ciências Farmacêuticas, Universidade de Sao Paulo, Sao Paulo, SP Brazil; 5 ThermoFisher Scientific, Sao Paulo, SP Brazil; 6 Departamento de Quimica Fundamental, Instituto de Quimica, Universidade de Sao Paulo, Sao Paulo, SP Brazil; 7 Departamento de Patologia, Universidade Federal de Sao Paulo, Sao Paulo, SP Brazil

## Abstract

We evaluated whether the excluded stomach (ES) after Roux-en-Y gastric bypass (RYGB) can represent a premalignant environment. Twenty obese women were prospectively submitted to double-balloon enteroscopy (DBE) with gastric juice and biopsy collection, before and 3 months after RYGB. We then evaluated morphological and molecular changes by combining endoscopic and histopathological analyses with an integrated untargeted metabolomics and transcriptomics multiplatform. Preoperatively, 16 women already presented with gastric histopathological alterations and an increased pH (≥4.0). These gastric abnormalities worsened after RYGB. A 90-fold increase in the concentration of bile acids was found in ES fluid, which also contained other metabolites commonly found in the intestinal environment, urine, and faeces. In addition, 135 genes were differentially expressed in ES tissue. Combined analysis of metabolic and gene expression data suggested that RYGB promoted activation of biological processes involved in local inflammation, bacteria overgrowth, and cell proliferation sustained by genes involved in carcinogenesis. Accumulated fluid in the ES appears to behave as a potential premalignant environment due to worsening inflammation and changing gene expression patterns that are favorable to the development of cancer. Considering that ES may remain for the rest of the patient’s life, long-term ES monitoring is therefore recommended for patients undergoing RYGB.

## Introduction

Bariatric surgeries, such as Roux-en-Y gastric by-pass (RYGB), are increasingly being performed to aid weight loss and favor metabolic effects, particularly in obese patients with type 2 diabetes (T2D)^1^. RYGB reduces gastric volume by defining a small proximal gastric pouch and excluding the remaining stomach (which is known as the excluded stomach [ES]) from the gastrointestinal tract, thereby making it a hard-to-reach ‘blind loop’^2^. The bypassed ES, remains in the patient’s body for life.

ES may harbour duodenal bile reflux following RYGB, and its accumulation has been associated with gastritis, intestinal metaplasia, and some rare cases of cancer^2–9^. In this scenario, duodenal bile reflux can be related to cancer risk as persistent alkaline reflux is accepted as a cause of gastric stump cancer due to Billroth II sub-total gastrectomy^8,9^.

We have previously shown that duodenal reflux affects 40% to 70% of RYGB patients and the remaining and excluded gastric chambers can present with elevated bacteria and fungi counts postoperatively^6^. We have also shown that patients undergoing RYGB surgery, with predominantly normal gastric endoscopy, can develop moderate or severe gastritis, atrophy, and intestinal metaplasia in the ES^5^. It is then possible that the ES can harbor duodenal bile reflux following RYGB, favoring the excessive growth of microorganisms, tissue injury, and subsequent malignance^9^.

After more than a half-century since the development of the RYGB technique, some authors have shown concerns about the potential inherent risk of this procedure for the development of ES cancer, and have highlighted some cases of the disease which were observed during the postoperative period^2,4–6,9^. Accordingly, surgeons from different countries with high rates of gastric cancer, such as Japan, Korea, and Chile, have suggested RYGB with resection of the ES^2,9^. In the present paper, we aimed to provide additional scientific data for this little-known condition and evaluated the general and molecular behavior of ES tissue, and its environment, after RYGB and its potential association with cancer development. To do this, we combined endoscopic and histopathological analyses with an integrated untargeted metabolomics and transcriptomics multiplatform.

## Methods

This study is part of the SURMetaGIT protocol^10^, registered at www.ClinicalTrials.gov (NCT01251016). The specific procedures involved were approved by the local institutional ethics board (Comissão de Ética para Análise de Projetos de Pesquisa - CAPPesq 1011/09) and were conducted according to the ethical standards of the World Medical Association’s Declaration of Helsinki.

### Study subjects and interventions

After obtaining informed consent, we studied 20 obese (body mass index ≥35 kg/m^2)^ adult women (age: 18–60 years), with T2D (fasting plasma glucose >126 mg/dL and hemoglobin A1c >6.5%) admitted for elective RYGB at the Gastrointestinal Surgery Division of the Hospital das Clinicas from University of Sao Paulo Medical School (HC-FMUSP). We recruited patients between 2011 and 2014. Patients were excluded if they suffered from type 1 diabetes, used insulin, had a *Helicobacter pylori* infection, suffered from thyroid or hepatic disease, or if they were currently participating, or had recently participated, on an interventional trial^10^.

All participants underwent RYGB, without silicon rings and with standardized biliary-pancreatic loops (50–60 cm) and feed handles (100–120 cm). Double-balloon enteroscopies were performed by an expert endoscopist (IR) before and 3 months after RYGB; these tests were carried out after 12-h fasting and 3–5 days abstinence from oral medications (except antihypertensive drugs). During the procedure, gastric macroscopic screening was carried out, with fluid reflux; local fluid and mucosal biopsies (10–15 mg) were also collected and frozen at −80 °C for subsequent untargeted metabolomic and transcriptomic analysis, respectively. A portion of the fluid was also used to measure pH, and a portion of the biopsies was formalin-fixed and embedded in paraffin for haematoxylin and eosin (H & E) staining and histopathological analysis. In each patient, the preoperative biopsy site was highlighted with India ink (SPOT; GI Supply, Camp Hill, PA) in order to ensure that postoperative collection occurred at the same location.

### Histopathology of the excluded stomach

Macroscopic alterations of the stomach fundus (pre-operatively) and ES (post- operatively) were screened during enteroscopy, such as gastritis, mucous lake and the presence of polyps. Gastritis was classified according to the Sydney grading system criteria^11^. Biopsies were also assessed for microscopic inflammation, mucosal atrophy, glandular dilation and intestinal metaplasia by two independent pathologists. Alterations were graded as follows: 0 = none; 1 = slight; 2 = moderate; and 3 = intense.

### Gastric fluid pH and metabolomic analysis

Samples of gastric fluid were thawed and homogenized for pH measurements, which involved gently dipping universal pH test strips (Merck, Darmstadt, Germany) into the fluid for colorimetric indication. Homogenized gastric fluid (200 µL) was pre-treated by protein precipitation with 600 µL cold methanol overnight (−20 °C). After centrifugation (15 min at 17,000 *g* and −4 °C), 250 µL supernatants were used as individual samples for metabolomic analyses. Quality control (QC) samples were prepared by mixing 200 µL of each supernatant; QCs were prepared by pooling equal volumes of all studied samples). A blank solution was prepared by using deionized water and the precipitation procedures described above. For liquid chromatography–mass spectrometry (LC-MS) analyses, samples were injected directly into the equipment, without any specific preparation. For gas chromatography–mass spectrometry (GC-MS) analyses, samples were evaporated to dryness and submitted to the derivatization protocol published previously by our group^12^.

LC-MS analyses (positive and negative modes) were performed on a Q Exactive Hybrid Quadrupole-Orbitrap Mass Spectrometer (Thermo Fisher Scientific, MA, U.S.A.) equipped with a nanoflow High Performance Liquid Chromatography system (Thermo Fisher Scientific, MA). GC-MS analyses were performed in a Gas Chromatography system coupled to a Single Quadrupole Mass Spectrometer (Agilent Technologies, CA, U.S.A.). Details of the specific methods and equipment parameters are presented in Supplementary Material S1.

Data processing was performed using XCMS software package (version 1.24.1). The parameters used for XCMS are described in Supplementary Material S1.

### Transcriptomic analysis of ES tissue

Transcriptomic analysis was carried out using the SURMetaGIT protocol^10^. In brief, following RNA extraction (RNeasy Plus kit-Qiagen, Germantown, MD), ES biopsies with an RNA concentration ≥100 ng/µL, and RNA integrity number (RIN) ≥7, were submitted for global expression analysis using the Human GeneChip 1.0 ST Array (Affymetrix, Inc., Santa Clara, CA). Significance of microarrays and rank product methods were analysed to select differentially expressed genes, using p < 0.05 (corrected for false discovery rate). Target analysis was also performed by real time quantitative polymerase chain reaction (RT-qPCR) to validate certain genes of interest which are potentially involved in carcinogenesis. We did this by using TaqMan gene expression assays (Thermo Fisher Scientific, Waltam, MA). β-actin was used as a reference gene.

### Statistical analysis

R software (version 3.1.3, 2015; R Core Team, Vienna, Austria) was used to report descriptive data as median (minimum–maximum). Pre- and postoperative data were compared using the nonparametric Mann–Whitney test.

For metabolomics data, we performed multivariate statistical analyses using Principal Component Analysis (PCA) and Partial Least Squares Discriminant Analysis (PLS- DA) models in SIMCA P+ software (12.0.1 version, Umetrics, CA, U.S.A.) and MetaboAnalyst 4.0, in which discriminant entities were selected according to Variable Importance Projection scores (VIP > 1). Univariate analyses were performed using the Mann–Whitney U test (*p*-value < 0.05), after checking data normality using Lilliefors test, performed in Statistica 13 software (StatSoft, OK, U.S.A.). Putative metabolites from LC-MS data were searched in the Human Metabolome Database, HMDB (http://www.hmdb.ca/), using [M + H]^+^, [2M + H]^+^ and [M + Na]^+^ adducts for positive ionization mode, and [M − H]^−^ and [M − 2H]^2−^ for negative mode. The metabolite identification for GC-MS data was performed using Fiehn RTL Library (Fiehn Lib) and the National Institute of Standards and Technology (NIST) database.

For tissue gene expression, the non-parametric Rank Products (RP) method was used to select Differentially Expressed Genes (DEGs), following a paired analysis design, p value < 0.05, and correction by False Discovery Rate (FDR). DEGs were analyzed using DAVID software (https://david.ncifcrf.gov) to identify biological processes and signal pathways potentially altered in ES and IPA (QIAGENInc; https://www.qiagenbioinformatics.com/products/ingenuitypathwayanakysis) to assess canonical pathways, networks and predictive regulatory networks^13^.

For all analyses, *p-*values < 0.05 were considered to be statistically significant. When a minimum sample was applied (n = 6 for gene expression; see Results section), the power of the study was >80% to detect differences in gene expression with an effect size ≥1.35-times the standard deviation (SD) of the difference.

## Results

### Sample descriptive data and flowchart

Main anthropometric and metabolic data are provided in Table 1. After 3 months of RYGB, all patients (mean age: 46.9 ± 6.2 years) experienced a significant reduction in body weight, body mass index (BMI), fasting blood glucose, and glycated haemoglobin values, as compared to the preoperative period.

**Table 1:** Descriptive data of obese women (n = 20) before and three months after Roux en-Y gastric bypass (RYGB).

Variables	Pre-operative	Postoperative	*p*-value*
Body weight (kg)	113.2 (83.5–143.6)	90.3 (68.2–113.7)	<0.001
Body mass index (kg/m^2^)	46.4 (37.1–7.5)	38.5 (30.3–45.5)	<0.001
Fasting glycaemia (mg/dL)	221.1 (77.0–321.0)	91.5 (75.0–153.0)	<0.001
Glycated haemoglobin (%)	9 (6–13)	6 (5–7)	<0.001

Data are expressed as median (minimum-maximum). *Mann–Whitney test.

Some surgical anatomical changes (intracavitary adhesions, fixed angulations, stomach stenosis, and extrinsic compression), along with increased peristalsis after intravenous sedation, precluded use of the enteroscopic device in the ES of 10 patients postoperatively. Furthermore, biopsy collection could not be safely performed in 4 of these 10 patients. Due to these complications, data from all samples (n = 20) were evaluated for macro and micro histological changes preoperatively. In order for each woman to serve as her own control, pH and molecular analyzes were performed only in those patients providing pre- and postoperative matching samples of ES juice obtained by successful enteroscopy (pH and metabolomics; n = 10) and those for which tissue had been successfully obtained by biopsy (transcriptomics; n = 6). Only 3 pre- and postoperative matching biopsies provided adequate RNA for microarray analysis, but target validation of gene expression was performed in all 6 samples. Supplementary Fig. S1 illustrates a flowchart of sample size for each of the studied variables.

### Macroscopic findings in the gastric tissue

At the macroscopic level, only 30% and 20% of patients presented with normal gastric mucosa pre- and postoperatively, respectively. Most patients (65%) already presented with gastritis preoperatively, including some with pangastritis (Table 2). Postoperatively, the incidence of gastritis remained high (80%) and progressed in intensity, with an almost 5-fold increased frequency of pangastritis. Furthermore, one case of intestinal metaplasia was observed and all patients presented with a green mucous fluid lake (Table 2).

**Table 2:** Macroscopic gastric histology and duodenal reflux of obese women before and 3 months after Roux en-Y gastric bypass.

Variable	Preoperative (n = 20)		Postoperative (n = 10)	
	n	%	n	%
Enanthematous gastritis	5	25	0	0
Erosive gastritis**	5	25	1	10
Atrophic gastritis	0	0	1	10
Enanthematous pangastritis*	3	15	7	70
Enanthematous pangastroduodenitis*	0	0	1	10
Presence of polyps	1	5	1	10
Intestinal metaplasia	0	0	1	10
Mucous green lake^♯^	0	0	10	100

Data expressed as number of patients. *Mild/Moderate; **High and flat; ^♯^Indicator of duodenal reflux.

### Gastric fluid pH and metabolite profile

**Figure 1:**
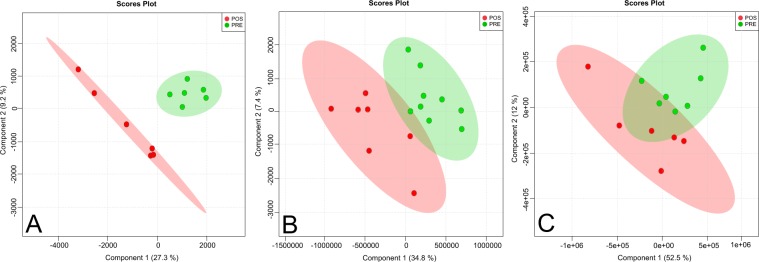
Partial Least Square Discriminant Analysis (PLS-DA) of the metabolomic profile of gastric fluid from the ES of obese women before and 3 months after RYGB. Analysis was performed in 10 patients using (**A**) GC-MS acquisition, (**B**) LC-MS positive mode acquisition, and (**C**) LC-MS negative mode acquisition. Pre, preoperative time point; Pos, 3-month postoperative time point.

**Figure 2:**
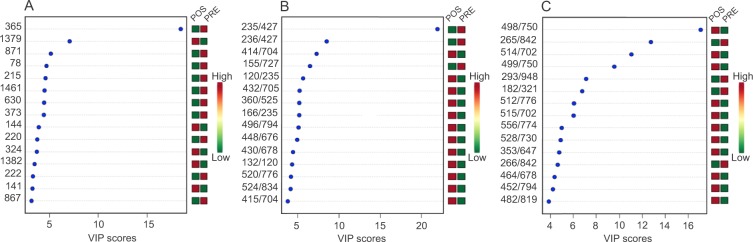
Metabolites that showed a higher contribution for separation in the metabolomics profile of gastric fluid from the ES of obese women between the periods before and 3 months after RYGB. Analysis was performed in 10 patients by using (**A**) GC-MS acquisition, (**B**) LC-MS positive mode acquisition, and (**C**) LC-MS negative mode acquisition. Unknown identities are represented as mass retention time. Identities known: 365, glucuronic acid. 1379, phosphoric acid. 1461, sucrose. 373, mannose. 496/794, docosapentanoyl carnitine. 520/776, LysoPC(18:2). 524/834, LysoPC(18:0). 514/702, taurocholic acid. 464/678, glycocholic acid. 482/819, litocholytaurine. Pre, preoperative time point; Pos, 3-month postoperative time point.

Eighty percent of patients (n = 10) presented with alkaline gastric fluid (pH ≥ 4.0) preoperatively (Supplementary Table S1) with a distinct global metabolomics profile when compared between pre- and postoperative periods, as showed by both PCA (Supplementary Fig. S2) and PLS-DA (Fig. 1) models. These distinct molecular patterns were more visible with GC-MS and LC-MS in positive mode platforms. Those metabolites with a higher contribution for group separation (periods) by PLS-DA are identified and highlighted in Fig. 2.

Based on the exact mass, retention time, and isotopic distribution, we identified 90 metabolites with different alterations between the pre- and postoperative period. Some of these included those participating in bile acid metabolism, phosphatidic acid cycle, and toxic metabolite conjugation; metabolites found in the urine and faeces; microbial metabolites; inflammatory mediators; and endocannabinoids (Table 3). Enriched metabolic pathways are shown in Supplementary Fig. S3.

**Table 3:** Metabolites undergoing significant changes in the gastric fluid of obese women 3 months after Roux en-Y gastric bypass.

Mass	Retention Time (min)	Metabolite	Fold Change	Biological Identities	Analytical Technique
408.2875	11.67	Trihidroxycholanoic acid/Allocholic acid/Cholic acid	51.3	Primary bile acid synthesis	LC-MS neg
374.2820	12.20	Hydroxycholenoate	7.87		LC-MS pos
515.2916	11.48	Taurohyocholate/taurocholic acid/taurallocholic acid	1.82		LC-MS pos
	11.71		1.61		LC-MS neg
532.3069	12.34	5b-Cyprinol sulphate	3.89	Bile alcohol	LC-MS neg
465.3090	11.30	Glycocholic acid	2.73	Primary bile acid	LC-MS pos
	11.31		1.24		LC-MS neg
449.3141	11.81	Chenodeoxycholic glycine conjugate/Glycoursodeoxycholic acid	3.07	Primary or secondary bile acid	LC-MS pos
433.3192	12.25	Lithocholic acid glycine conjugate	6.17	Secondary bile acid	LC-MS pos
	12.26		1.94		LC-MS neg
483.3018	13.64	Lithocholytaurine	2.45		LC-MS neg
499.2967	12.41	Tauroursodeoxycholic acid	2.80		LC-MS neg
390.2770	14.29	Hydroxyoxocholanoate (ketholithocolic acid)	0.54		LC-MS pos
529.2709	11.16	Glycochenodeoxycholic acid 3-sulfate	2.63	Sulphate bile acid	LC-MS neg
563.2586	12.67	Taurolithocholic acid 3-sulfate	1.46		LC-MS neg
304.2402	13.72	Arachidonic acid	1.67 2.04	Inflammatory mediators	LC-MS pos LC-MS neg
481.3168	12.92	LysoPC(15:0)	7.73		LC-MS pos
493.3168	12.74	LysoPC(16:1)	8.04		LC-MS pos
495.3324	13.23	LysoPC(16:0)	2.24		LC-MS pos
509.3481	13.55	LysoPC(17:0)	7.16		LC-MS pos
517.3168	12.55	LysoPC(18:3)	15.4		LC-MS pos
519.3324 521.3481	12.93 13.42	LysoPC(18:2) LysoPC(18:1)	7.63 3.88		LC-MS pos
					LC-MS pos
523.3637	13.91	LysoPC(18:0)	3.12		LC-MS pos
541.3168	12.51	LysoPC(20:5)	25.8		LC-MS pos
547.3637	13.00	LysoPC(20:2)	20.4		LC-MS pos
567.3324	12.83	LysoPC(22:6)	12.6		LC-MS pos
392.2327	16.12	Phosphatidic acid (16:0)	2.12	Turnover membrane and Phosphatidic acid cycle	LC-MS pos
	17.54		1.68		LC-MS neg
416.2327	16.69	Phosphatidic acid (18:2)	10.1		LC-MS neg
180.0633	18.89	*myo*-inositol	1.88		GC-MS
572.2961	16.37	1-Palmitoylglycerophosphoinositol	0.81		LC-MS neg
600.3274	18.58	Stearoylglycerophosphoinositol	0.81		LC-MS neg
620.2961	8.59	Arachidonoylglycerophosphoinositol	0.02		LC-MS neg
686.4910	8.91	DG(20:5n3/0:0/22:6n3)	0.42		LC-MS neg
714.5223	9.19	DG(20:5n6/0:0/22:6n3)	0.47		LC-MS neg
327.3137	12.22	Stearoylethanolamide	2.85	Endocannabinoid	LC-MS pos
194.0426	16.89	Glucuronic acid	0.02	Toxic metabolite conjugation	GC-MS
132.0575	6.82	Atropaldehyde	5.50	Drug metabolite (found in the urine)	LC-MS neg
433.2100	8.68	Dextrorphan O-glucuronide	2.76		LC-MS neg
551.2519	12.14	Endoxifen O-glucuronide	1.16		LC-MS neg
440.2344	13.70	Tirofiban	1.13		LC-MS neg
592.3260	9.53	Mesobilirubinogen	5.61	Bilirubin catabolism (found in the urine and faeces)	LC-MS pos
594.3417	9.57	L-Stercobilin	4.69		LC-MS pos
466.3116	11.29	Cholesterol sulphate	3.73	Steroid biosynthesis (found in the urine)	LC-MS pos
	11.30		1.24		LC-MS neg
318.2558	14.38	Pregnanolone	14.6		LC-MS pos
320.2715	14.78	Pregnanediol	47.9		LC-MS pos
464.2410	14.30	Dehydroepiandrosterone 3-glucuronide	0.27		LC-MS pos
164.0684	15.21	Fucose	0.15	Microbial metabolites: fermentation of non-digestible polysaccharides and proteins (SCFAs)	GC-MS
150.0528	14.66	Ribose/Lyxose (pentose monosaccharide)	0.13		GC-MS
152.0684	15.14	Arabitol/Ribitol/Xylitol (sugar alcohol)	0.17		GC-MS
270.2558	16.97	C16 methyl palmitate	3.71		GC-MS
298.2871	18.88	C18 methyl stearate	1.88		GC-MS
161.0840	13.14	Tryptophanol	4.79		LC-MS pos
214.1317	1.81	Dethiobiotin	8.53	Microbial metabolites: vitamin synthesis	LC-MS pos
278.1266	5.35	N1-(alpha-D-ribosyl)-5,6-Dimethyl-benzimidazole	3.02		LC-MS pos
246.1215	1.97	L-beta-Aspartyl-l-leucine	1.26	Microbial metabolites: cholesterol synthesis	LC-MS neg
414.3497	11.79	Hydroxymethyl-cholestadienol	18.6		LC-MS pos
384.3392	16.57	Cholesterol synthesis intermediates	10.3		LC -MS pos
				Warburg effect	
175.0956	17.28	Citrulline	2.14	Nitric oxide synthesis	GC-MS
180.0633	16.93	Mannose/Glucose/Allose (aldohexose)	0.95	Glycolysis	GC-MS
157.0738	11.71	Methylcrotonylglycine	3.54	Mitochondrial damage	LC-MS pos
385.2828	11.07	Hydroxytetradecenoyl-L-carnitine	10.9	Acylcarnitines	LC-MS pos
421.3192	11.45	Linolenylcarnitine	2.94		LC-MS pos
423.3348	11.92	Acylcarnitine C18:2	9.08		LC-MS pos
425.3505	12.18	Acylcarnitine C18:1	9.24		LC-MS pos
275.1368	8.24	Glutaryl-L-carnitine	1.13		LC-MS neg
285.1940	9.04	Octenoyl-L-carnitine	4.90		LC-MS pos
287.2096	9.71	Octanoyl-L-carnitine	3.95		LC-MS pos
313.2253	10.22	9-Decenoylcarnitine	6.83		LC-MS pos
315.2409	10.60	Decanoyl-L-carnitine	7.86		LC-MS pos
341.2566	10.90	Dodecenoyl-L-carnitine	5.07		LC-MS pos
471.3348	12.76	Cervonylcarnitine	8.04		LC-MS pos
473.3505	13.23	Docosapentaenoylcarnitine/Clupadonylcarnitine	2.22		LC-MS pos
256.2402	18.14	Palmitic acid	28.0	Fatty acid synthesis and uptake	GC-MS
282.2558	19.71	Oleic acid	11.4		GC-MS
280.2402	19.67	Linoleic acid	11.3		GC-MS
	13.76		1.54		LC-MS neg
97.9768	9.49	Phosphoric acid	36.3	Membrane phospholipid synthesis	GC-MS
188.1160	4.76	N-acetyl-lysine	3.08	Active gene transcription process (hystone acetylation)	LC-MS pos

Metabolites were identified by GC-MS and LC-MS (VIP score > 1, and *p*-value < 0.05). LysoPC, lysophosphatidylcholine; DG, diacylglycerol.

### Microscopic histology and transcriptomics in the gastric tissue

**Figure 3:**
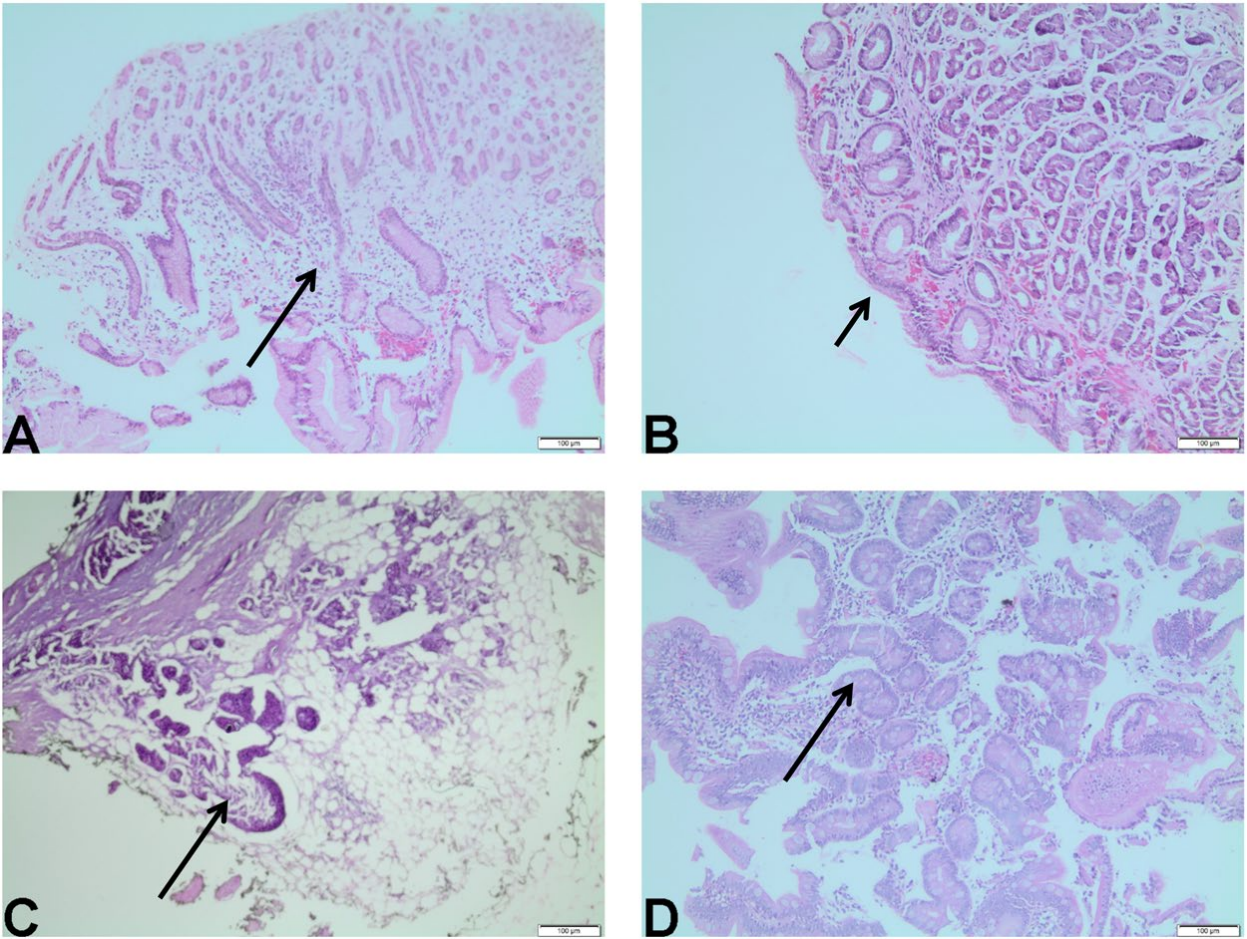
Representative histological findings in the ES of obese women before and 3 months after RYGB. (**A**) gastritis; (**B**) atrophic gastritis; (**C**) glandular dilatation; (**D**) intestinal metaplasia.

At the microscopic level, representative histopathological findings included gastritis, atrophic gastritis, glandular dilatation, and intestinal metaplasia (Fig. 3). Prior to RYGB, most patients already showed some grade of inflammation and mucosal atrophy with gastritis (Table 4). We also observed glandular dilatation and intestinal metaplasia foci (Table 4). Following surgery, inflammation and mucosal atrophy remained the most frequent histological change (Table 4), but the intensity of these alterations increased among patients with pre- and postoperative matching biopsies and one patient progressed with new intestinal metaplasia (Table 5).

**Table 4:** Gastric microscopic histopathology of obese women before and 3 months after Roux en-Y gastric bypass.

Variable	Preoperative (n = 20)		Postoperative (n = 6)	
	n	%	n	%
Inflammation	18	90	6	100
Atrophy	13	65	5	83
Glandular dilation	8	40	4	67
Intestinal metaplasia	2	10	1	17

**Table 5:** Evolution of the altered gastric histology grade of obese women, before and 3 months after Roux en-Y gastric bypass.

Patient’ code	Inflammation		Atrophy		Glandular dilatation		Intestinal metaplasia	
	T0	T1	T0	T1	T0	T1	T0	T1
A	1	**2**	1	**2**	1	**2**	0	**1**
B	0	**1**	0	**2**	1	**2**	0	0
C	1	**2**	0	**1**	0	0	0	0
D	2	**3**	1	1	0	0	0	0
E	2	2	2	**3**	2	2	0	0
F	1	1	0	0	0	**1**	0	0

Bold numbers highlight the evolution in degree of inflammation, atrophy, glandular dilation and intestinal metaplasia 3 months after the surgery (T1), compared to the preoperative period (T0). 0, absent; 1, slight grade; 2, moderate grade; 3, intense grade.

Global microarray analysis identified 135 differentially expressed genes (DEGs) after RYGB (38 upregulated genes and 97 downregulated genes; p < 0.05). Based on these, functional enrichment, using the DAVID software internal data base, identified processes involved in inflammation and immune response to microorganisms as the main biological pathways increased in ES tissue after RYGB, representing 50% of the changes induced by surgery (Table 6). Processes involved in increased cell proliferation and histone acetylation, necrosis, and apoptosis, together with those related to cell metabolism changes, represented 20% of the local postoperative metabolic alterations (Table 6).

**Table 6:** More prominent biological processes in the ES tissue after RYGB, according to the DAVID database.

Biological process	Count	%	p value	Benjamini
Imune response	9	10,1	4,3E-4	2,3E-1
Chemotaxis	5	5,6	1,9E-3	4,4E-1
Chemokine-mediated signaling pathway	4	4,5	3,5E-3	5,1E-1
Cell-cell signaling	6	6,7	4,7E-3	5,1E-1
defense response to virus	5	5,6	5,5E-3	4,9E-1
Signal transduction	12	13,5	1,0E-2	6,5E-1
Response to virus	4	4,5	1,2E-2	6,4E-1
Negative regulation of osteoblast differentiation	3	3,4	1,2E-2	6,1E-1
Negative regulation of endopeptidase activity	4	4,5	1,5E-2	6,5E-1
Regulation of oligodendrocyte progenitor proliferation	2	2,2	2,1E-2	7,3E-1
Positive regulation of cAMP metabolic process	2	2,2	2,5E-2	7,6E-1
Positive regulation of energy homeostasis	2	2,2	2,5E-2	7,6E-1
O-glycan processing	3	3,4	2,7E-2	7,6E-1
Glucose metabolic process	3	3,4	3,4E-2	8,0E-1
T cell chemotaxis	2	2,2	3,4E-2	7,8E-1
Striated muscle cell differentiation	2	2,2	4,2E-2	8,3E-1
Positive regulation of T cell migration	2	2,2	4,2E-2	8,3E-1
Regulation of cell proliferation	4	4,5	4,5E-2	8,3E-1
Positive regulation of cAMP-mediated signaling	2	2,2	5,0E-2	8,4 E-1
Regulation of necrotic cell death	2	2,2	5,0E-2	8,4 E-1
Negative regulation of smooth muscle cell migration	2	2,2	5,8E-2	8,7E-1
Peptidyl-tyrosine dephosphorylation	3	3,4	6,7E-2	8,9E-1
Negative regulation of peptidase activity	2	2,2	7,0E-2	8,90E-1
Positive regulation of histone acetylation	2	2,2	7,4E-2	9,0E-1
Positive regulation of leukocyte chemotaxis	2	2,2	7,4E-2	9,0E-1
Negative regulation of smoothened signaling pathway	2	2,2	7,8E-2	9,0E-1
Negative regulation of growth	2	2,2	7,8E-2	9,0E-1
Inflammatory response	5	5,6	8,0E-2	8,9E-1
Cellular response to lipopolysaccharide	3	3,4	8,5E-2	9,0E-1

Accordingly, signaling pathways highlighted by the KEGG database as showing the most activation after RYGB also included those related to response to inflammation and microorganisms, together with pathways directly associated with cancer (Supplementary Table S2).

**Figure 4:**
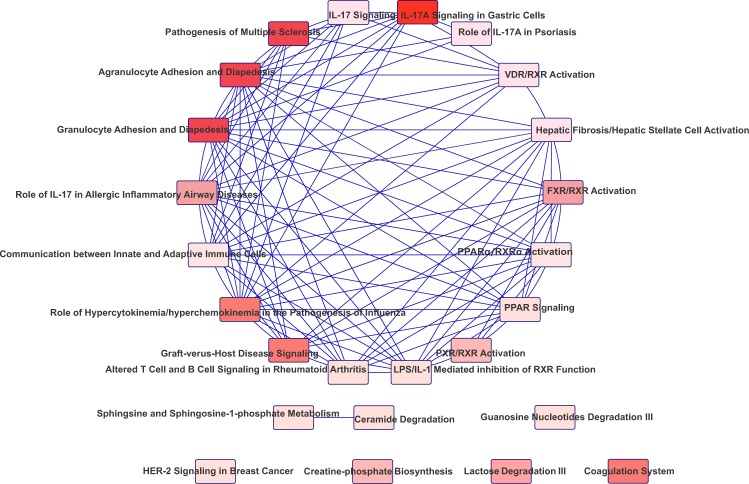
Canonical pathways in the ES tissue activated by RYGB. Data were analyzed via the use of IPA (QIAGEN Inc.; https://www.qiagenbioinformatics.com/products/ingenuitypathway-analysis).

**Figure 5:**
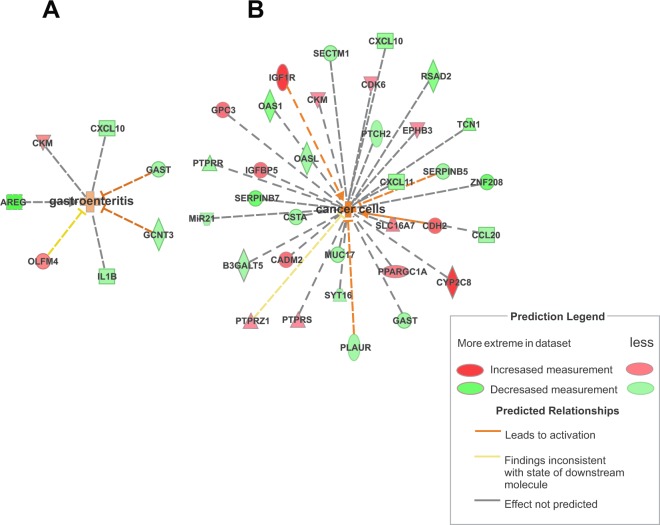
Gene interaction networks in the ES tissue activated by RYGB, showing (**A**) gastroenteritis and (**B**) carcinogenesis as biological networks activated by the surgery. Data were analyzed by IPA (QIAGEN Inc.; https://www.qiagenbioinformatics.com/products/ingenuitypathway-analysis).

**Figure 6:**
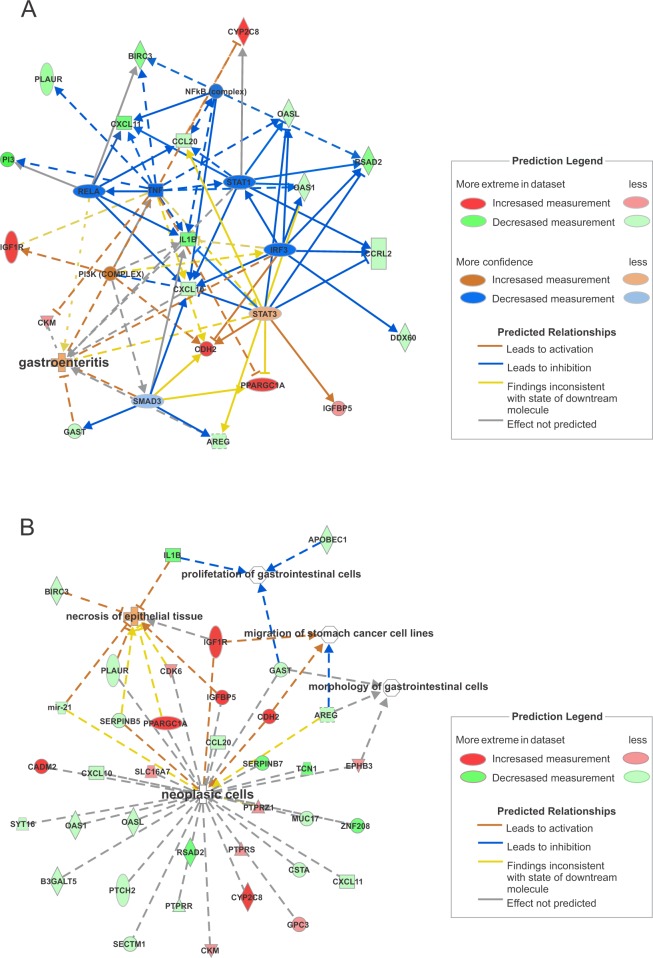
Gene interaction network associated with the predictive activation of (**A**) infectious inflammation (gastroenteritis) and (**B**) epithelial cell necrosis and cell proliferation towards neoplasia after RYGB. Data were analyzed by IPA (QIAGEN Inc., https://www.qiagenbioinformatics.com/products/ingenuitypathway- analysis).

**Figure 7:**
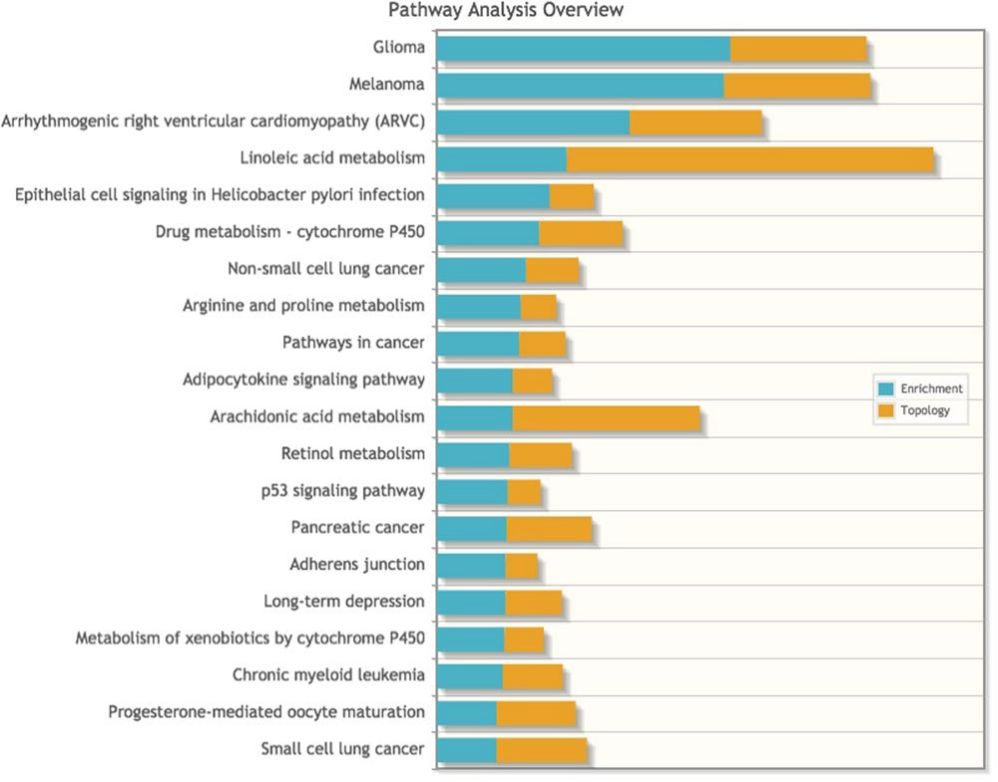
Joint analysis, combining our transcriptomic data with our metabolomis data, highlighted pathways involved in cancer development, inflammation, and immune response to microorganisms.

IPA software identified the 24 most connected canonical pathways that were significantly changed in the ES tissue after RYGB. These also included pathways associated with inflammation, in response to bile and microorganisms, immune tolerance, and cancer development (Fig. 4). Some DEGs involved directly or indirectly with the identified canonical pathways were validated by quantitative reverse transcription polymerase chain reaction (RT-qPCR, Supplementary Table S3). The analysis of gene interaction networks highlighted biological processes involved in infectious inflammation (gastroenteritis) and cancer development (Fig. 5). Additional predictive analysis of regulatory genes showed that the activation of gastroenteritis by RYGB was independent of classical nuclear factor kappa B (NFkB) activation and tumor necrosis factor alpha (TNF-α) expression (Fig. 6A). Instead, the set of processes significantly altered after RYGB indicated activated epithelial cell death/necrosis processes, while cell repopulation or tissue regeneration occurred at the expense of changes in the expression of genes involved in carcinogenesis (Fig. 6B). Joint analysis, combining our transcriptomic data with the metabolomic data highlighted pathways involved in cancer development, inflammation, and immune response to microorganisms (Fig. 7).

## Discussion

In this study, we identified abnormal gastric histology in obese women that worsened after RYGB in parallel to an increased alkaline duodenal reflux into the ES, which was rich in bile acids (BA) and metabolites common to the intestinal environment (urine and faeces). Our molecular analyzes suggested the existence of a potential premalignant environment created by the fluid accumulating in the bypassed organ, which caused inflammation to worsen and promoted the expression of genes that may favor carcinogenesis in the long-term. Considering that the ES will remain for the rest of the patient’s life, potentially for 30–50 years, this tissue needs to be monitored periodically.

Bile-rich duodenal reflux has previously been directly related to postoperative gastric mucosal damage^2,4,5,7,9^. Accordingly, our patients presented with a predominant increase of BA in the gastric fluid contained in the ES, including primary, secondary, conjugated, and sulphated BA. It is possible that the increased amounts of abdominal fat in obese women physically favors a light preoperative duodenal gastric reflux. The changes in gastrointestinal anatomy induced after RYGB appears to increase the intensity of this reflux and BA accumulation with more intense consequences on the normal architecture of the ES, regardless of body weight loss.

A typical example of the inflammatory potential of BA is cholestatic disease. This can be triggered by the lipophilic characteristics of BA, mainly conjugated, by promoting cell-membrane damage via activation of the *phospholipase A2* (PLA2) and *interleukin 17* *A* (IL-17A) pathway^14^. PLA2 activation is involved in the production of the pro-inflammatory eicosanoid precursor arachidonic acid (AA) and lysophosphatidylcholine (LysoPC), while IL-17A favors inflammation by increasing neutrophil recruitment and proinflammatory cytokine synthesis through pathways independent of TNF-α and NFkB activation^14,15^. In our study, we observed increased levels of AA and LysoPC in the ES along with IL-17A gene activation, suggesting that the accumulation of conjugated BA may have activated inflammatory pathways, contributing to the progression of tissue inflammatory alterations.

Complementary metabolic data supported the non-physiological accumulation of primary BA at the ES. In epithelial cells, primary BA promotes sulphation of accumulated bile by activating nuclear receptors such as the farnesoid X receptor (FXR), pregnane X receptor (PXR), vitamin D receptor (VDR), and peroxisome proliferator-activated receptor coativator 1 alpha (PPARGC1A). Sulphated BA are less toxic compounds than non-sulphated BA and is more suitable for faecal and urinary excretion^15^. We observed predictive activation of these nuclear receptors, together with increased sulphated BA, indicative of the activation of biological pathways which deal with toxic bile accumulation. It is worth noting that even sulphated BA may lose its protective role and become highly inflammatory when accumulated instead of excreted^16^.

In our study, some drugs and metabolites that use bile as the excretion route had also accumulated in the ES, as suggested by the increase in metabolites often found in the urine and faeces after RYGB^17–19^. Accumulation of these products may have clinically relevant consequences. The biliary enterohepatic cycle may result in ‘false positive’ systemic hepatic feedback, while non-excreted and accumulated metabolites may become co-participants of bile-induced tissue inflammation in the ES.

On the other hand, some BA, mainly secondary BA, can exert anti-inflammatory effects by activating the G-protein coupled bile acid receptor 5 (TGR5). This receptor can reduce the production of pro-inflammatory cytokines (TNF-α, IL-1β and IL-6) induced by lipopolysaccharides (LPS) in neutrophils and macrophages via NFkB inhibition^20^. Moreover, TGR5 can activate endocannabinoid pathways and enhance their immunosuppressive action by the synthesis of stearoylethanolamide, a substrate of the cytochrome P450 family 2 subfamily C member 8 (CYP2C8)^21–24^. However, TGR5 activation is associated with a certain immunological tolerance, which is modestly effective in reducing inflammation^21,25^. In the present study, increased secondary BA and stearoylethanolamide in the gastric fluid, combined with the increased tissue expression of *CYP2C8*, are indicative of the activation of TGR5 in the ES. Consistent with this, levels of the *IL-1β* gene were significantly reduced and predictive gene analysis revealed that the inflammation observed in the ES occurred independently of TNF-α and NFκB (other TGR5 targets).

However, considering its immunosuppressive role, TGR5 may favor immunological tolerance and bacterial colonization, especially by bacteria involved in secondary BA synthesis^20,21,25^. In support of this hypothesis, we observed an increase in phosphatidic acid, a membrane lipid essential for bacterial immune tolerance that can be generated by the activation of TGR5^21^.

Other molecular markers of tolerance to microorganisms were altered in RYGB-ES, such as the olfactomedim 4 (OLFM4) gene and transmembrane 1 gene (SECTM1). The gastric increase of OLFM4 is related to *H*. *pylori* colonization by decreasing NFκB activity and inhibiting inflammation, while the reduced expression of SECTM1 may be considered as a potential marker of immunosuppressive response to constant exposure to LPS^26,27^.

In tumors, increased OLFM4 expression is associated with differentiation, staging, metastasis, and poor prognosis, suggesting potential clinical value as a tumor marker in the early stages of carcinogenesis^26^. Changes in the expression of SECTM1 and OLFM4, as well as in the activity of TGR5, play a role in the immunological tolerance to cancer cells and the colonization of tissues by bacteria^26,27^. We also found significantly reduced expression of radical S-adenosyl methionine domain containing 2 (RSAD2) and increased expression levels of protein tyrosine phosphatase, type S receptor (PTPRS) in ES tissue. Both genes encode proteins involved in virus-immune tolerance, suggesting immunological tolerance to the virus after RYGB^28,29^.

Once in the tissue, microorganisms have an important impact on metabolism by complementing human enzymatic activities and promoting health or disease^30^. Gram-negative bacteria (e.g. *Escherichia coli*) are more resistant to the lipophilic characteristics of BA and can grow in environments with high concentrations of bile^31^. In the bile-enriched environment of the ES, a potentially higher amount of Gram-negative resistant bacteria could act on the primary BA to produce its secondary derivatives^20,32^. Under anaerobic conditions, the predominant secondary BA formed is ursodeoxycholic. This, and its conjugated derivative, tauroursodeoxycholic (TUDCA), is hydrophilic, less toxic, and anti apoptotic^32,33^. The increase of TUDCA, and decrease of ketolitocolic acid, observed by us in the ES points to a predominantly anaerobic environment and the participation of Gram-negative bacteria to control the toxicity of accumulated bile after the procedure.

Under such anaerobic conditions, microorganisms may synthesize vitamins and ferment undigested carbohydrates and amino acids derived from dietary or host-derived proteins to short-chain fatty acids (SCFAs), such as butyrate and propanoate, in order to supply energy and cholesterol and thus regulate lipid metabolism. Gases, such as hydrogen and carbon dioxide are consequences of fermentation. The removal of these gases allows fermentation to continue. Specifically, methanogenesis uses hydrogen and carbon dioxide to produce methanol and transesterify fatty acids. This process is relevant for intestinal health by replacing the inflammatory characteristics of saturated fatty acids with anti-inflammatory functions^30–36^.

In the present study, we observed increased metabolism and synthesis of vitamins, oxidation of SCFAs, and metabolism of propanoate as methyl palmitate and methyl stearate (transesterified lipids); these findings support the presence of anaerobic bacteria in the RYGB-ES. Similarly, the observed increase in tryptophanol, hydroxymethyl-cholestadienol, and cholesterol are indicative of proteolytic activity. *Myxobacteria*, a type of Gram-negative anaerobic bacteria, exclusively synthesize hydroxymethyl-cholestadienol into cholesterol from leucine^36^.

In particular, the typically low stomach pH values (pH 1–3) seem important for the appropriate production of mucin (MUC) which acts as a barrier for pathogenic bacteria and controls inflammation^37,38^. We found a significant reduction of *MUC17*, *GCNT3* (glucosaminyl (N-acetyl) transferase 3, mucin type) and *B3GALT5* (beta-1,3- galactosyltransferase 5) expression following RYGB-ES; all of these factors are involved in the production of mucin^37–42^. We also observed an increase of intellectin 1/omentin (*ITLN1*) expression, which is responsible for the mucosal secretion of galactose-linked lecithin that participates in the recognition of pathogenic bacteria^42^. *ITLN-1* overexpression has been reported in intestinal metaplasia and may be a marker of intestinal-type gastric tumors^41^. In our study, mucin expression decreased following RYGB-ES, together with an increase in *ITLN-1* and galactose metabolism, thereby favoring potential colonization by microorganisms.

Postoperative inflammation, the excessive growth of microorganisms, and the generation of reactive oxygen species (ROS), together with a reduction in mucin, are likely to act as aggressive factors in ES epithelial tissue, inducing cell death with subsequent signaling for repopulation. Notably, *ITLN1* is predominantly expressed in the gut where it can stimulate epithelial cell survival through Akt activation; following RYGB-ES, we observed increased levels of *ITLN* but reduced levels of the mitogenic gastric cell factor GAST. Our molecular findings suggest that stress-induced tissue regeneration in ES may progress towards intestinal metaplasia, and be considered as a pre-tumor lesion^41,42^.

Successful stress-induced regeneration depends on cell proliferation. In our study, the increased expression of insulin-like growth factor 1 receptor (*IGF1R*), insulin-like growth factor binding protein 5 (*IGFBP5*), glypican-3 (*GPC3*), and cyclin dependent kinase 6 (*CDK6*), together with a decrease in *s*erpin family B (SERPINB) 5 and 7 suggest that the RYGB-ES cells are proliferating and that the tissue extracellular matrix is remodeling to accommodate new cells. In breast and stomach cancers, the increased expression of *IGF1R*, *GPC3*, and *IGFBP5* genes is associated with tumor progression and metastasis^43–46^. Moreover, extracellular matrix remodeling occurs when the expression of *SERPINB5* and 7 is reduced, favoring cellular proliferation and migration and, in cases of cancer, invasion and metastasis^47^.

Cell repopulation involves the fine control of cell positioning and adhesion in tissue; an imbalance in these processes are often observed in tumors^48,49^. In our transcriptomic analysis, the significant increase of Eph receptor B3 (*EphB3*), in conjunction with cadherin 2 (*CDH2)*, suggests tissue regeneration, positional control, and cell adhesion in the ES. *EphB3* is often expressed in the intestinal epithelium and functions as an important agent in cell repositioning during tissue regeneration^48^. This can positively regulate the expression of cadherins such as *CDH2*, thus increasing cell adhesion^48^. However, an increase of EphB3 expression in tissues that do not normally express this gene, such as the lung, appears to promote oncogenic transformation and tumor development^48^.

Any process involving tissue remodeling and proliferation can be downregulated by protein phosphatases (PTPs), including protein tyrosine phosphatase, receptor type R (PTPRR). PTPRR can inhibit the *IGF1R* or *Eph* pathways by increasing its degradation^50^. In the RYGB-ES, the observed significant reduction in PTPRR expression suggests less degradation of both molecules, thus prolonging the proliferation signaling triggered by these receptors. Clinical and experimental studies have recognized that the epigenetic silencing of *PTPRR* is an early event in colorectal tumorigenesis^50^.

Epigenetic events control gene expression and play an important role in carcinogenesis. Epigenetic silencing (mainly of tumor suppressor genes), a result of DNA methylation and histone deacetylation, is the most frequent epigenetic event observed in tumors. However, in our transcriptomic and metabolomic analysis, the increase in N-acetyl-lysine (metabolite from histone acetylation; Table 3), along with positive regulation of the histone acetylation pathway, observed by DAVID analysis (Table 6), suggests an active gene transcripton process in ES-cells and not a global mechanism of gene silencing or DNA methylation. Interestingly both events may be related to carcinogenesis since epigenetic events are transient. A previous experimental study, using breast cells cultured with chemical carcinogens, demonstrated that during malignant transformation, the silencing or increase in gene expression depended on the dose and time of exposure to the carcinogens. In addition, when the chemical carcinogen was removed from the culture medium, the gene transcription process was regulated in a manner similar to the control cells^51^. This means that highly proliferative environments exposed to toxic agents are susceptible to both epigenetic events and may subsequently facilitate genetic mutations if the stressful stimulus is not controlled or removed^51^. In the ES-microenvironment, the immunological tolerance acquired with the continuation of stress (reflux), suggested by our molecular data, could favor genetic instability and impair the elimination of mutated cells. In the long term, the metabolic and genetic changes promoted by postoperative reflux may result in irreversible mutations and make the ES a fertile environment for cancer development. In our transcriptomic analysis, we observed a non-significant decrease in the expression of the mutL homolog 1 (*MLH1*) gene early after RYGB. This gene encodes a protein that detects and repairs DNA damage during cell proliferation^52^. It is possible that in the long-term, the downregulation of *MLH1* becomes significant and results in the accumulation of DNA mutations with increased risk for cancer development.

For clinical cancer development, genetic mutations must contribute to and maintain clonal expansion. This includes, but is not limited to: (i) proliferative advantages, and (ii) disorders in cell cycle control and DNA repair, or even the absence of restrictive proliferation signals. When present, these changes are considered shortcuts to the development of cancer. However, for complete malignant transformation, a third component, which binds (i) and (ii) appears to be mandatory: the metabolic reprogramming of cells, including their microenvironment^53^.

Activation of the mammalian target of rapamycin (mTOR) protein complex is common in inflammatory processes, immune tolerance, and cancer, and is considered to lin these processes together^54^. mTOR regulates the expression of genes and signaling pathways involved in cell metabolism, in an attempt to prevent the interruption of immune and proliferative functions owing to a lack of energy and oxygen. In order to maintain energy delivery, mTOR enhances pathways involved in rapid ATP synthesis (via the Warburg effect), while stimulating mitochondrial biogenesis via the expression of *PPARGC1A*. The end products of glycolysis are shared between resident cells by the monocarboxylate transporter 2 (*MCT2*), a transporter encoded by the solute carrier family 16-member 7 (*SLC16A7*) gene to avoid interruptions in metabolic flow. mTOR also increases the synthesis of cholesterol and fatty acids, at the expense of decreased beta-oxidation, mainly to synthesize membranes and their lipid rafts. These membrane microdomains harbor and activate proliferative (*IGF-1R* and *GPC-3*) and immunological (*TGR5*) signaling receptors, as well as the concentrated production of phosphatidic acid. mTOR is especially activated by the unsaturated chains of phosphatidic acid^21,54^. Our data showed compatible changes of these genes and metabolites, suggesting that ES fluid after RYGB activates mTOR for the maintenance of local inflammatory, tolerant, and proliferative conditions

Taken together, genetic and metabolic alterations observed in the RYGB-ES were similar to those occurring in carcinogenesis, although it is unlikely that these arose from gene mutations, considering the short analysis period after surgery (3 months). Nevertheless, the hostile environment of the ES highlighted by our data (with absence of food along with biliary reflux rich in intestinal content, decreased mucins, increased pH, excessive microorganism growth and increased ROS production) may enhance inflammation and activate immunological tolerance in the long-term, thus hindering the elimination of mutated cells.

It is possible that the diagnosis of cancer in patients undergoing RYGB-ES has been underestimated, as endoscopic access to this anatomical portion in the postoperative period is difficult^2,4–6,9^. This clinical challenge represents the most relevant scientific limitation of our study: data relating to postoperative histological, metabolomics, and mainly transcriptomics were obtained from ≤50% of the total number of patients recruited. This also prevented us from obtaining an adequate amount of ES-tissue with which to assess functional and mechanistic data related to gene expression, which was based solely at the transcriptional level. However, gene expression can be influenced by environmental factors and our metabolomic analysis identified significant alterations in the ES environment. Furthermore, the observed set of DEGs shared common biological pathways, in which activation/inhibition was consistent with the identified environmental changes. Another limitation of our study was the fact that our analyses only involved obese women. This, however, ensured that we had a more homogeneous sample, as gender may have influenced gene expression. In addition, most bariatric patients in our institution are women.

The development and application of alternative methods for cancer screening in the RYGB-ES, such as medical imaging, should be strongly encouraged. Alternatively, surgeons should be invited to reflect on the relevance of conserving the excluded stomach as part of the RYGB technique. Our molecular data supports the need for some action in these directions, by highlighting the fact that RYGB can generate a pre-malignant environment in ES early after the procedure. Some of our findings are very relevant at this point, such as the local presence of 5-ciprinol sulphate, a toxic bile alcohol present in the adult phase only under specific dysfunctional conditions, such as inflammation and carcinogenesis in the liver^55^. Because carcinogenesis is usually a long-term process, it may be possible that reports relating to RYGB-ES cancer will increase over time. It is probable that surgery does not behave as a pre-neoplasm lesion for gastric cancer but rather exposes the ES-RYGB to a detrimental environment with a potential risk for cancer development.

## Supplementary information

Supplementary Info
