# Supplementary material for: Potential premalignant status of gastric portion excluded after Roux en-Y gastric bypass in obese women: A pilot study
Source: Sci Rep. 2019 Apr 3;9:5582. doi: 10.1038/s41598-019-42082-4 (PMC6447527; doi:10.1038/s41598-019-42082-4)
Supplement: Supplementary file 1 — Supplementary Info [file 41598_2019_42082_MOESM1_ESM.pdf]

Potential premalignant status of gastric portion excluded after Roux en-Y gastric bypass in obese women: A pilot study

Graziela Rosa Ravacci[1], Robson Ishida[2], Raquel Suzana Torrinhas[1], Priscila Sala[1], Natasha Mendonça Machado[1], Danielle Cristina Fonseca[1], Gisele Canuto[3], Ernani Pinto[4], Viviane Nascimento[5], Marina Tavares[6], Paulo Sakai[2], Joel Faintuch[2], Marco Aurelio Santo[2], Eduardo Guimarães Hourneaux Moura[2], Ricardo Artigiani Neto[7], Angela Flávia Logullo[7] and Dan Linetzky Waitzberg[1].

[1]Departamento de Gastroenterologia, Laboratorio Metanutri (LIM35), Faculdade de Medicina FMUSP, Universidade de Sao Paulo, Sao Paulo, SP, BR; [2]Hospital das Clinicas HCFMUSP, Faculdade de Medicina, Universidade de Sao Paulo, Sao Paulo, SP, BR; [3]Departamento de Quimica Analitica, Instituto de Quimica, Universidade Federal da Bahia, Salvador, BA, BR; [4]Faculdade de Ciências Farmacêuticas, Universidade de Sao Paulo, Sao Paulo, SP,BR; [5]Thermo Fisher Scientific, Sao Paulo, SP, BR; [6]Departamento de Quimica Fundamental, Instituto de Quimica, Universidade de Sao Paulo, Sao Paulo, SP, BR; [7]Departamento de Patologia, Universidade Federal de Sao Paulo, Sao Paulo, SP, BR;

Corresponding Author: Graziela Ravacci. Av. Dr. Arnaldo, 455, 2° andar, sala 2208 – Cerqueira César Post code: 01246-903, São Paulo – SP, Brazil; Phone / Fax: +55 11 3061-7459. E-mail: grazielametanutri@gmail.com

SUPPLEMENTARY FILES

Materials S1 – Metabolomics Methods

Reagents. Acetonitrile (LC-MS grade), formic acid (FA, LC-MS grade) heptane (MS grade), N,O-bis(trimethylsilyl)trifluoroacetamide (BSTFA) with 1% (v/v) trimethylchlorosilane (TMCS), *O*-methoxyamine, and pyridine were purchased from Sigma-Aldrich (Germany). Methanol (LC-MS grade) was acquired from JT Baker (Mexico).

Three untargeted metabolomic analytical platforms were used, focusing on the profiles of low weight (m/z < 1500) ionizable molecules which were present in at least 50% of the sample of each group.

Metabolomics sample preparation. Sample pre-treatment involved protein precipitation using 200 µL of homogenized gastric fluid and 600 µL of methanol. The content was mixed in an ultrasound bath (Quimis, Brazil) using a mixing vortex (Quimis, Brazil) and kept overnight at -20°C. The precipitate was then separated by centrifugation (Universal 320R, Hettich, Germany) for 15 min at 17,000*g* at -4°C, and 250 µL of the supernatant was used for metabolomic analyses. Quality Control (QC) samples, a pool of all studied samples, were prepared by mixing 200 µL of each supernatant. A blank solution was prepared, using deionized water and cold methanol, according to the procedures described above. For LC-MS analyses, samples were directly injected into the system. For GC-MS analyses, blank, samples, and QCs were evaporated to dryness using a SpeedVac (Thermo Scientific SPD1010, U.S.A.) at 40°C and submitted to a derivatization protocol[1]. In brief, 10 µL of *O*-methoxyamine (15 mg mL^{-1}) in pyridine was added to each of the dried samples. The mixed solution was maintained in the dark at room temperature for 90 min, after which 10 µL of BSTFA + 1% TMCS (v/v) was added. The mixed solution was maintained in a thermostatic bath (ECO SILVER RE 1225,

Lauda, Brazil) at 40°C for 30 min. The derivatized samples were finally resuspended in 100 μL of heptane prior to GC-MS analyses.

LC-MS acquisition. LC-MS analysis was performed on a nanoflow High-Performance Liquid Chromatography system (ThermoFisher Scientific, MA, U.S.A.) coupled to a Q Exactive Hybrid Quadrupole-Orbitrap Mass Spectrometer (ThermoFisher Scientific, MA, U.S.A.) via electrospray ionization. A reversed phase C18 column (Hypersil Gold, 100 mm × 2.1 mm i.d, 1.9 μm particles; ThermoFisher Scientific) maintained at 40°C was used. The mobile phase consisted of (A) deionized water acidified with 0.1% (v/v) FA, and (B) acetonitrile acidified with 0.1% (v/v) FA. The separation method was adapted from Saric *et al.* (2012).[2] The gradient elution, for positive and negative modes of ionization, was: 0.1% B for 2 min, 0.1–25% B for 6 min, 25–80% B for 10 min, 80–90% B for 12 min, and 90–99.9% B for 21 min. The flow rate was 400 μL/min. The MS acquisition was in profile mode and operated at 3.2 kV in the positive mode and 2.6 kV in the negative mode. The capillary temperature was 350°C and the spray voltage was 4.00 kV; sheath gas was 30 units and auxiliary gas flow rate was 10 units. Full scan data in both ionization modes used 35,000 mass resolution, and a scan range of *m/z* 100 to 1500 was chosen. The system was operated using Thermo Xcalibur software (Thermo Scientific).

GC-MS acquisition. GC-MS analysis was performed in a Gas Chromatography system coupled to a Single Quadrupole Mass Spectrometer (Agilent Technologies, CA, U.S.A.). An HP-5 ms column (30 m length, 0.25 mm i.d., with a 0.25 μm film composed of 95% dimethyl and 5% diphenylpolysiloxane; Agilent Technologies) was used. The separation method was as previously used,[1] in which He at 1.0 mL/min flow rate was the carrier gas, and the GC injector was maintained at 250°C. Samples were injected with a 1:10 split, using He at 10 mL/min. The temperature gradient was as follows: initial oven temperature of 60°C maintained for 1 min, increased to 300°C at 10°C/min rate. The run time was 25 min. The detector transfer line,

filament source, and the quadrupole were maintained at 290, 230, and 150°C, respectively. The electron ionization source used -70 eV energy, and the MS was operated in scan mode in the range of 50–600 *m/z*. The system was operated using Mass Hunter B.07.01 software (Agilent Technologies).

Data treatment and statistical analysis. Metabolomics data processing was performed using the XCMS software package (version 1.24.1) [3] running on R platform (3.2.3 version, R Foundation for Statistical Computing), using a matched filter method for peak detection, peak alignment, and retention time correction. GC-MS raw data files were converted into *.mzData using Qualitative Analysis Mass Hunter software (B.05.00, Agilent Technologies), and LC-MS raw data files (positive and negative modes) were converted into *.mzXML using ProteoWizard software.[4] For GC-MS data, the XCMS parameters were: fwhm (full width at half maximum of model peak) = 3, snthresh (signal-to-noise cutoff) = 1, max (maximum number of groups to identify in a single *m/z* slice) = 40; first grouping with bw (bandwidth) = 4 and second grouping with bw = 2, mzwid (width of overlapping *m/z* slices) = 0.5, and minfrac (minimum fraction of samples necessary in one group to be a valid group) = 0.5. Other parameters were kept at default values. For LC-MS data, XCMS parameters were: first and second grouping with bw = 10 and mzwid = 0.0025, peak picking and retention time correction (retcor) were used at default values. For GC and LC data treatment, after second grouping, the fillPeaks tool was used to integrate areas of missing values.

Statistical analysis was carried out using multivariate and univariate methods. In multivariate analyses, PCA (Principal Component Analysis) and PLS-DA (Partial Least Squares Discriminant Analysis) were performed in SIMCA P+ software (12.0.1 version, Umetrics, CA, U.S.A.) and the MetaboAnalyst platform.[5] Discriminant entities were selected based on the Variable Importance Projection (VIP score > 1.0) from PLS-DA models in SIMCA P+ software. Univariate analysis was performed in Statistica 13 software (StatSoft, OK, U.S.A.)

using Mann–Whitney U test (p-value < 0.05) after checking data normality with the Lilliefors test, and False Discovery Rate (FDR < 0.05) using Benjamini and Hochberg procedure.

Metabolite annotation. Putative metabolite identification for LC-MS data (positive and negative modes) was performed by searching for significant mass-to-charge ratios (*m/z*) in Human Metabolome Database, HMDB (http://www.hmdb.ca/). For positive ionization mode, $[M+H]^+$, $[2M+H]^+$, and $[M+Na]^+$ were selected as possible adducts, and for negative ionization mode, $[M-H]^-$, $[2M-H]^-$, and $[M-2H]^{2-}$ were chosen;5 ppm was used for both modes as the maximum allowed error. Metabolite identification for GC-MS data was performed using AMDIS 2.69 (Automated Mass Spectral Deconvolution and Identification System) software with Fiehn RTL Library (FiehnLib).[6] Retention time and mass spectra fragmentation pattern, after retention index and retention time correction, were used to peak assignment, greater than 700 and 85, respectively.

References

1. Canuto, G.A.B. et al. New insights into the mechanistic action of methyldehydrodieugenol B towards Leishmania (L.) infantum via a multiplatform based untarget metabolomics approach. Metabolomics 13-56 (2017)
2. Saric, J. et al. Systematic evaluation of extraction methods for multiplatform-based metabotyping: application to the Fasciola hepatica metabolome. Anal Chem 84, 6963–72 (2012)
3. Smith, C.A., Want, E.J., Tong, G.C., Abagyan R. & Siuzdak G. XCMS: processing mass spectrometry data for metabolite profiling using nonlinear peak alignment, matching, and identification. Anal Chem 78, 779–87 (2006)
4. Chambers, M.C. et al. A cross-platform toolkit for mass spectrometry and proteomics. Nat Biotechnol 30, 918–20 (2012)
5. Xia, J. et al. MetaboAnalyst 2.0--a comprehensive server for metabolomic data analysis. Nucleic Acids Res 40, W127–33 (2012)
6. Kind, T. et al. FiehnLib: mass spectral and retention index libraries for metabolomics based on quadrupole and time-of-flight gas chromatography/mass spectrometry. Anal Chem 81, 10038–48 (2009)

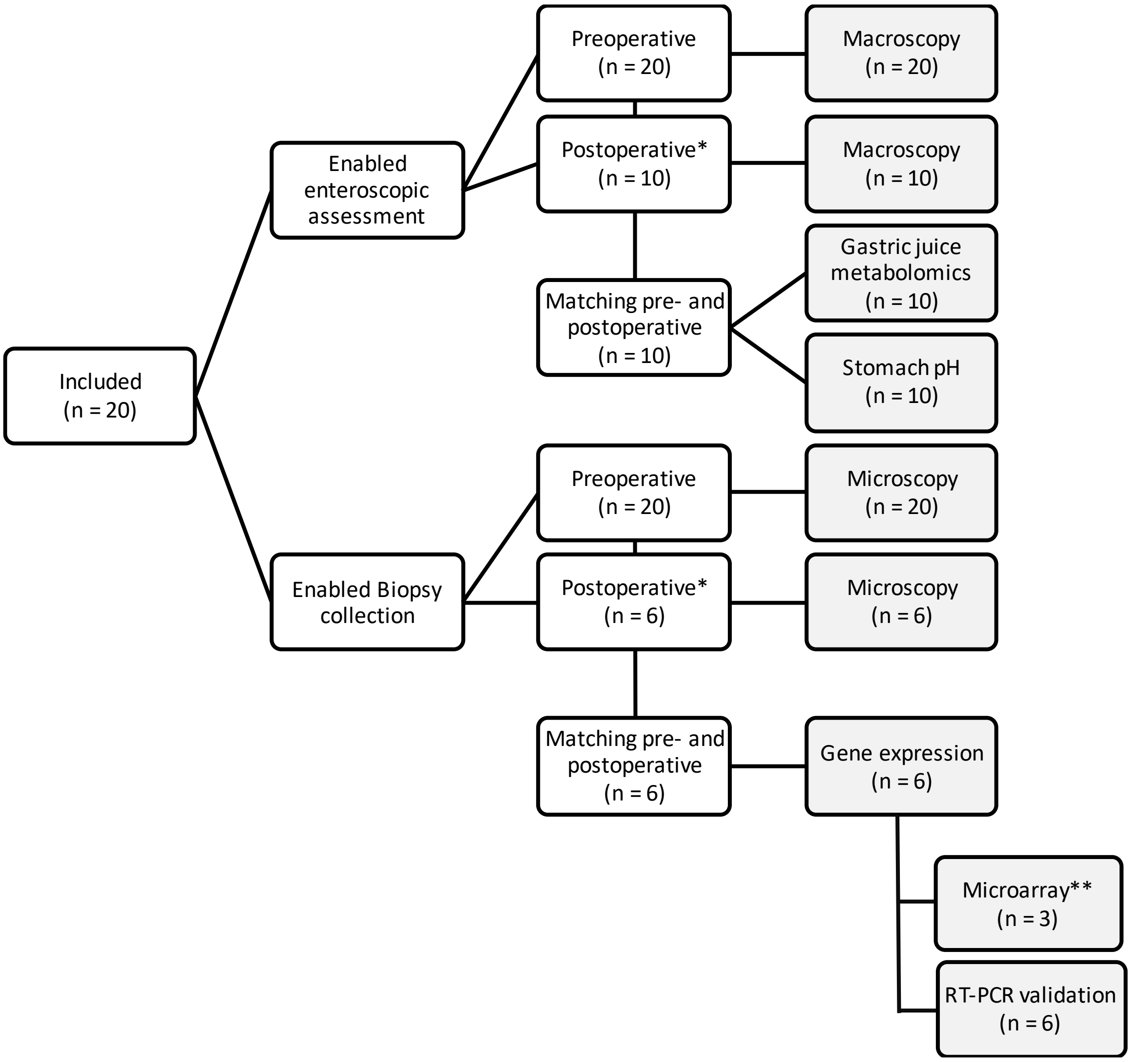

Figure S1. Flowchart showing patient assessments. Factors impairing overall sample analysis: *Intracavitary adhesions and fixed angulations; anatomical changes, such as stenosis and extrinsic compression; increased peristalsis after intravenous sedation. **Inadequate amount and/or quality of extracted RNA. RT-PCR, Reverse transcription polymerase chain reaction

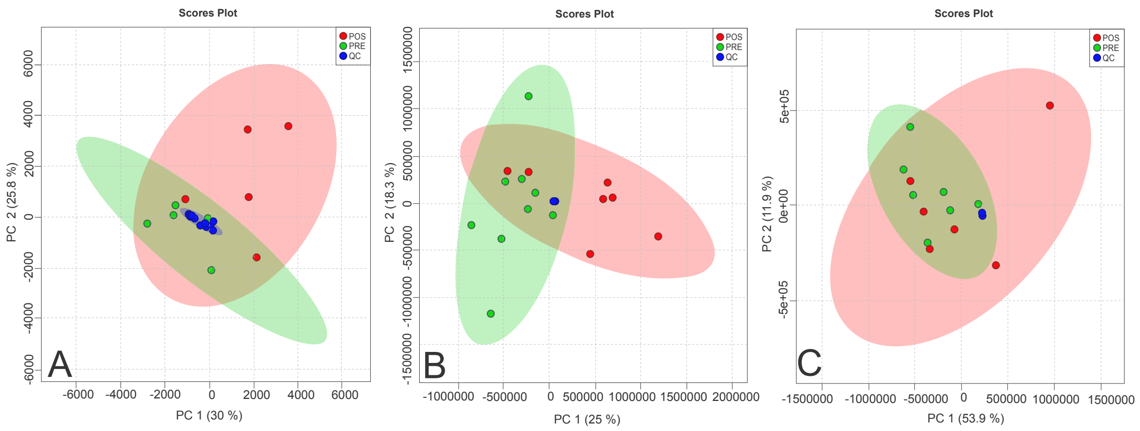

Figure S2. Principal Component Analysis (PCA) of the metabolomic profile of gastric fluid from the ES of obese women before and 3 months after RYGB. Analysis was performed in 10 patients by using (A) GC-MS acquisition, (B) LC-MS positive mode acquisition, and (C) LC-MS negative mode acquisition. Pre, preoperative time point; Pos, 3-month postoperative time point; QC, quality control.

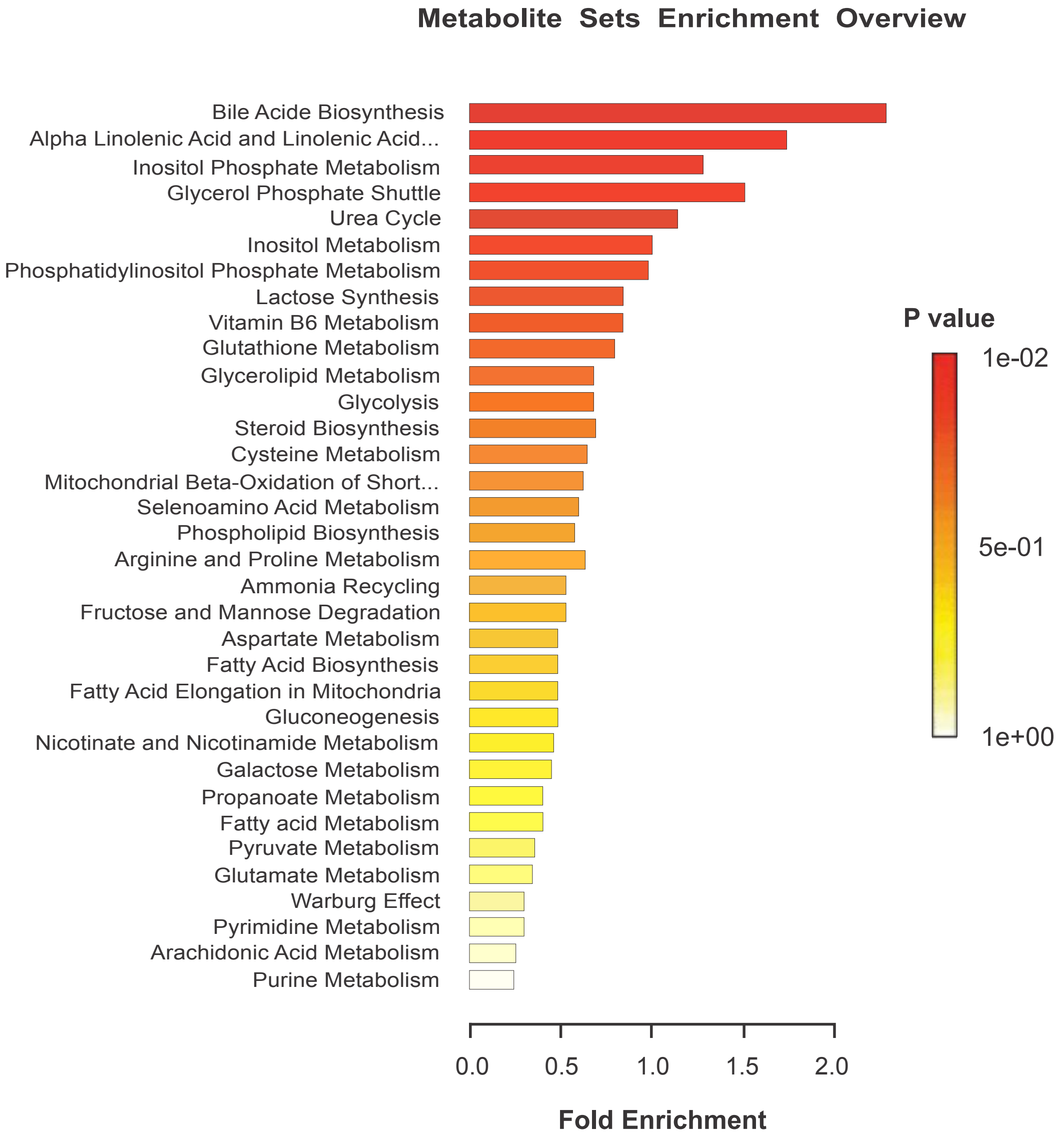

Figure S3. Enriched metabolic pathways identified in ES-gastric fluid after RYGB . Analyses were performed from metabolites found to be significantly altered after RYGB by using the Metaboanalyst 4.0 software.

Table S1. Gastric pH of obese women before and 3 months after Roux en-Y gastric bypass.

Patient's code	Preoperative	Postoperative
A	1-2	2-3
B	5-6	6-7
C	4	7-8
D	8-9	4
E	8-9	7-8
F	7-8	4
G	4	6-7
H	5-6	7-8
I	2-3	2-3
J	5-6	4

Table S2. Top signaling pathways activated in the ES tissue, 3 months after RYGB, according to the KEGG database.

**Representative pathways**	**Count**	**%**	**p value**	**Benjamini**
Influenza A	5	5,6	1,0E-2	6,8E-1
TNF signaling pathway	4	4,5	1,5E-2	5,7E-1
Complement and coagulation cascades	3	3,4	4,5E-2	8,2E-1
Rheumatoid arthritis	3	3,4	6,9E-2	8,7E-1
Proteoglycans in cancer	4	4,5	4,7E-2	8,2E-1
Toll-like receptor signaling pathway	3	3,4	9,5E-2	8,5E-1

Table S3. Differentially expression genes involved directly or indirectly with the canonical pathway identified in the ES after RYGB.

GENE	FOLD CHANGE
PPARGC1A	**1.4 (M)**
IL-1β	**1.2 (M)**
CYP2C8	**1.8 (M) 2.8 (V)**
OLFM4	**1.4 (M)**
SECTM1	**1.2 (M)**
RSAD2	**1.6 (M)**
PTPRS	**1.0 (M)**
MUC17	**1.2 (M)**
GCNT3	**1.2 (M) 1.3 (V)**
B3GALT5	**1.4 (M)**
GAST	**1.5 (M)**
ITLN1	**1.7 (M) 3.3 (V)**
IGFR1	**1.1 (M) 2.1 (V)**
GPC3	**1.3 (M) 1.2 (V)**
IGFBP5	**1.4 (M) 1.8 (V)**
CDK6	**1.0 (M)**
EphB3	**1.0 (M)**
CDH2	**1.4 (M) 1.6 (V)**
SERPINB5	**1.2 (M)**
SERPINB7	**2.2 (M)**
PTPRR	**1.3 (M)**
SLC16A7	**1.2 (M)**
*MLH1	**0.2 (M) 0.8 (V)**

Upregulated genes are in red, while downregulated genes are in green ($p < 0.05$). M, microarray ($n = 3$); V, RT-PCR validation ($n = 6$). *not significant
